# Ubiquitous Sirt1 overexpression is associated with preserved cardiac diastolic function and mitochondrial respiratory capacity in mice on a high-fat diet

**DOI:** 10.1016/j.metop.2026.100503

**Published:** 2026-09-29

**Authors:** Andrea Schrepper, Michael Schwarzer

**Affiliations:** Department of Cardiothoracic Surgery, Jena University Hospital, Friedrich Schiller University of Jena, Jena, Germany

**Keywords:** Sirt1 overexpression, High-fat diet, Diastolic dysfunction, Insulin-Akt signaling

## Abstract

**Background:**

Chronic high-fat diet (HFD) induces obesity, insulin resistance, and mitochondrial dysfunction, serving as a critical driver of diabetic cardiomyopathy. In turn, the NAD + -dependent deacetylase Sirt1 regulates metabolic flexibility and cellular survival, making it a promising therapeutic target to counteract this diet-induced cardiovascular damage.

**Methods:**

Here, we investigated the impact of a moderate, systemic Sirt1 overexpression (∼3-fold via a BAC transgenic mouse model; Sirt1^OE^) on HFD-induced metabolic and cardiac remodeling. Sirt1^OE^ and wild-type (WT) male mice were fed a HFD for 8 weeks. Whole-body glucose homeostasis, cardiac function, cardiac insulin signaling, and complex I-supported mitochondrial state 3 respiration (via Clark electrode) were assessed.

**Results:**

HFD-fed WT mice developed obesity, fasting hyperglycemia, impaired glucose tolerance, isolated diastolic dysfunction (pre-HFpEF phenotype), and cardiac insulin resistance, characterized by blunted Akt phosphorylation. In Sirt1^OE^ mice, HFD-induced diastolic dysfunction was significantly mitigated, and maximal mitochondrial respiratory capacity remained preserved. This protection was associated with a selectively maintained downstream Akt (Ser473) activation despite upstream insulin receptor inhibition. On a systemic level, Sirt1^OE^ mice exhibited accelerated glucose clearance and a marked reduction in fat mass accumulation.

**Conclusion:**

Moderate systemic Sirt1 overexpression protects against HFD-induced myocardial mitochondrial respiratory dysfunction and partially mitigates diastolic dysfunction. This cardioprotection is apparently cell-autonomously supported by the preservation of downstream insulin-Akt signaling, though partly facilitated by a reduced systemic adiposity and metabolic load. Strategies augmenting Sirt1 pathways warrant further investigation as therapeutic approaches against metabolic cardiomyopathies.

## 1 Introduction

A chronic high-fat diet (HFD) exerts detrimental effects on nutritional status and life expectancy, imposing immense economic burdens on global healthcare systems. Obesity, in particular, is heavily associated with dyslipidemia, systemic hypertension, and insulin resistance, and has emerged as a major independent risk factor for specific cardiac complications. Characteristically, metabolic stress triggers myocardial remodeling that leads to isolated diastolic dysfunction with a preserved ejection fraction (the HFpEF phenotype) [1]. At the cellular level, obesity-related cardiac insulin resistance is mechanistically linked to impaired mitochondrial respiratory capacity [2,3]. This metabolic derangement restrains the primary pathway of ATP generation, disrupts mitochondrial fusion and fission dynamics, and suppresses organelle biogenesis [4]. Together, this nexus of mitochondrial decay and the collapse of downstream insulin signaling cascades - particularly the canonical Akt survival pathway - is widely considered a major contributor behind HFD-related cardiomyopathies [[5], [6], [7]].

Recently, Sirt1, an NAD^+^-dependent protein deacetylase, has been identified as a metabolic sensor that pairs fluctuations in nutrient availability with cellular survival pathways, chiefly regulating insulin sensitivity and mitochondrial homeostasis [8]. Under low-nutrient conditions such as starvation, intracellular NAD^+^ levels rise, stimulating Sirt1 activity, whereas nutrient excess under a chronic HFD depletes the NAD^+^ pool, precipitating a decline in Sirt1 function [9,10]. Beyond its role in metabolic adaptation, Sirt1 regulates critical cellular processes, including longevity [11], apoptosis [12], DNA repair [13], and inflammatory responses [14,15]. Notably, mice featuring a moderate, systemic overexpression of Sirt1 (∼3-fold) are protected against diet-induced metabolic syndrome [16,17]. This indicates that variations in Sirt1 signaling are not merely a passive epiphenomenon of metabolic crises, but that Sirt1 acts as a decisive upstream regulator in the pathology of metabolic diseases. However, it remains poorly understood whether the regulatory effects of Sirt1 on specific mitochondrial respiratory complexes underlie these protective outcomes, and how direct Sirt1 elevation shapes the development of HFD-induced diastolic dysfunction.

To elucidate the role of Sirt1 in metabolic cardiomyopathy and dissect the underlying molecular mechanisms, we subjected a well-characterized natural-promoter BAC transgenic mouse model (Sirt1^OE^) and wild-type (WT) controls to 8 weeks of HFD. We subsequently performed an integrated assessment of systemic glucose homeostasis, in vivo cardiac diastolic performance, and complex-specific mitochondrial respiration capacity. Our investigation aims to clarify whether a targeted elevation of Sirt1 can rescue the myocardial signaling architecture and preserve mitochondrial fitness in the face of severe chronic metabolic stress.

## 2 Material & methods

### 2.1 Animal handling and diet intervention

All animal experiments conformed to the “Guide for the Care and Use of Laboratory Animals” published by the National Institutes of Health (NIH Publication no. 85-23, revised 1996), and the experimental protocols were approved by the local authorities (Thüringer Landesamt für Verbraucherschutz, approval ID 02-081/14). To ensure animal welfare, all mice were monitored closely by weekly inspections and weight measurements.

Sirt1 overexpressing mice (Sirt1^OE^) were a kind gift of Manuel Serrano, Madrid (Spain). Briefly, Sirt1^OE^ mice were previously generated by pronuclear microinjection of a bacterial artificial chromosome (BAC) carrying a large genomic segment (174 kb) encoding the entire Sirt1 gene and the adjacent gene Dnajc12 as detailed elsewhere [17]. Importantly, Sirt1^OE^ mice show a moderate Sirt1 overexpression across different tissues (∼3-fold increase).

Male transgenic Sirt1 mice and non-transgenic wild-type (WT) littermates were generated by heterozygous breeding on a C57BL/6J background. Only male offspring were utilized to prevent sex-specific confounders; female littermates were assigned to independent breeding/experimental protocols. Mice were housed in groups of up to 5 in individually ventilated cages (IVC) at 21 °C with light-dark cycles of 12 h and received water and food *ad libitum*. Male mice were randomly assigned to either standard grain-based chow (standard diet, SD - V1534, ssniff Spezialdiäten GmbH) or high-fat diet (HFD - D12492, ssniff Spezialdiäten GmbH; 60% energy from fat – lard) feeding regimens, starting at two months of age and lasting for 8 weeks (Fig. 1A and B).

**Fig. 1:**
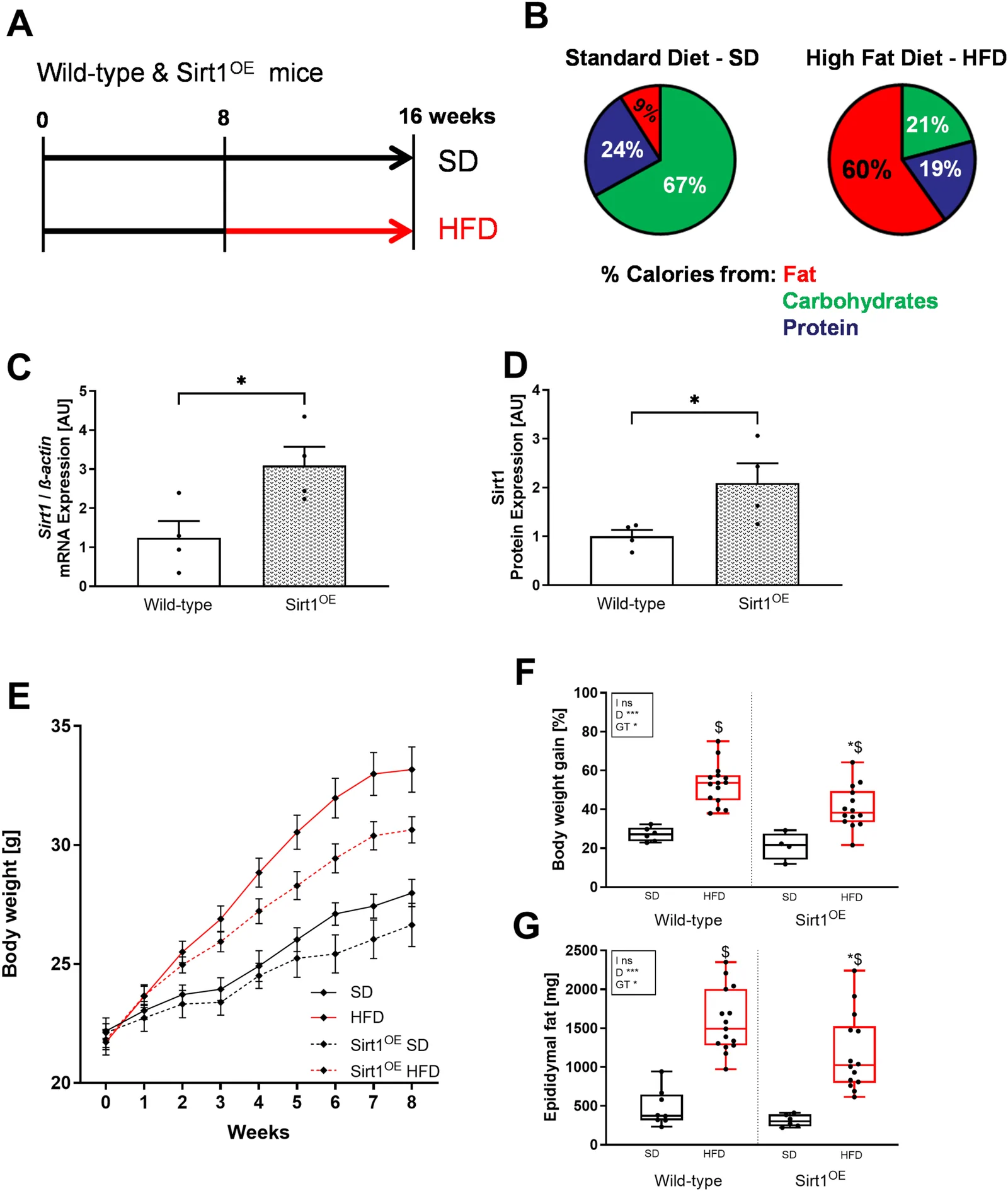
**High-fat diet-induced gain in body mass is attenuated in Sirt1^OE^ mice.** (**A**) Study design. (**B**) Composition of diets. (**C**) Sirt1 mRNA expression in hearts of Sirt1^OE^ and WT mice (n = 4). (**D**) Sirt1 protein level in hearts of Sirt1^OE^ mice and WT mice (n = 4). (**E**) Development of body weight in g in the course of 8 weeks HFD (n = 5-15). (**F**) Gain of body mass in % (n = 4-15). (**G**) Gain in epididymal fat mass in mg (n = 6-15). Data are shown as mean ± SEM or as box-and-whisker plots with individual data points. Appropriate statistical test was used: Student's t-test *P < 0.05 and ***P < 0.001 for comparisons as indicated in panel **C** and **D** or two-way ANOVA - $ *P* < 0.05 vs. SD of same genotype, *P < 0.05 vs. wild-type of same diet in panel **F** and **G**. SD, standard diet; HFD, high-fat diet; Sirt1^OE^, Sirt1 transgenic mice; I, interaction; D, diet; GT, genotype.

#### 2.1.1 Sequential study design and sample Sizes

Non-invasive phenotypic and functional parameters (e.g., body weight, tolerance tests and echocardiography) were measured across a main part of the study cohort to ensure adequate power for overall phenotypic characterization. For invasive, specialized downstream biochemical (e.g., Western blotting, PCR) and organelle-level assays (isolated cardiac mitochondria), pre-defined sub-cohorts of littermates were utilized based on tissue processing capacity and predefined power considerations. There were no animal exclusions, dropouts, or protocol deaths during the study. Following functional in vivo profiling, animals were euthanized for terminal organ harvest. Biochemical (Western blot, qPCR) and organelle-level functional assays (isolated cardiac mitochondria) were performed on representative sub-cohorts of littermates to balance tissue availability and processing capacity. All investigators performing functional assays were blinded to genotype and diet during data acquisition and analysis.

### 2.2 Glucose and insulin tolerance tests

Tolerance tests were routinely performed in the morning at 8 a.m. to avoid circadian variations as described elsewhere [[18], [19], [20]]. Briefly, the mice were fasted for 6 h prior to a tolerance test by using a food trap. To minimize acute handling stress and ensure accurate, reproducible intraperitoneal injections, mice were subjected to brief, transient sedation using isoflurane (5% induction) lasting less than 1 min immediately prior to injection. Animals recovered full consciousness within seconds after administration. For glucose and insulin tolerance tests (GTT, ITT), a single bolus of 2 g/kg BW glucose or a single bolus of 1 U/kg BW insulin were administered intraperitoneally (*i.p.*), respectively. One drop of peripheral blood from the tail tip of awake mice was sampled at 15, 30, 60, 90 and 120 min after injection, and blood glucose levels were assessed using a hand-hold glucometer (Freestyle mini, Abbott, Germany). Glucose and insulin tolerance tests were performed at least 2 days apart.

### 2.3 Echocardiography

Echocardiography was performed prior to termination of the experiment, i.e., 8 weeks after starting the SD and HFD intervention. Initial anesthesia was induced with 5% isoflurane in 100% oxygen in an induction chamber. Maintenance was delivered via a fitted nose cone 1% isoflurane). Following the procedure, mice recovered completely within 3-5 min. This protocol was executed identically across all test sessions and experimental cohorts. Chests were shaved and mice were positioned in supine position on a heated platform with integrated ECG electrodes and a rectal temperature probe to maintain core body temperature strictly at 37.0 ± 0.5 °C. A Vevo 770 with a 30 MHz phased array transducer for examinations (RMV 707b; Visual Sonics, Canada) was used to assess two-dimensional short-axis views of the left ventricle at the level of papillary muscles. Two-dimensional-guided M-mode tracings were recorded with a sweep speed of 100 mm/s [21]. The following parameters were obtained: heart rate (HR), ejection fraction (EF), left ventricular wall thickness, and end-diastolic dimensions (LVEDD). The mitral inflow pattern was assessed from the apical 4-chamber view with the use of pulsed-wave Doppler and the sample volume placed at the mitral leaflet tips. Mitral inflow Doppler measurements included early ventricular filling velocity (E Wave), late active ventricular filling velocity (A Wave), and the deceleration time (DT) of the E Wave. Parameters derived of the mitral inflow Doppler included E/DT and E/A. For all M-mode and Doppler parameters, measurements were taken across three consecutive cardiac cycles and averaged to yield a single representative value per biological replicate.

### 2.4 Organ harvesting

Immediately after echocardiography, mice were sacrificed via cervical dislocation. Hearts and other organs were excised, weighed, and processed or stored. Hearts for mitochondrial isolation were rinsed in cardioplegic solution (180 mM KCl, 10 mM EDTA, pH7.4) and stored on ice. Tissue samples for Western blot and PCR analyses were rinsed using 0.9% NaCl solution and immediately shock-frozen in liquid nitrogen and stored at −80 °C.

To assess acute cardiac insulin signaling, sub-cohorts of male mice were subjected to acute insulin stimulation (1 U/kg *i.p.*, 5 min) or saline control (basal) prior to tissue collection. Each experimental sample represents a distinct biological replicate from an individual animal.

### 2.5 Isolation of mitochondria and determination of respiratory capacity

After breaking up cardiac tissue, mitochondria were isolated following a differential centrifugation procedure according to Palmer et al. [22] with a modified Chappel-Perry buffer (180 mM KCl, 10 mM EDTA, 0.5% BSA, pH 7.4). Isolated mitochondria were kept on ice in KME buffer (100 mM KCl, 50 mM MOPS and 0.5 mM EGTA, pH 7.4). Mitochondrial protein concentrations were determined by the Bradford method using BSA as a standard. Mitochondrial citrate synthase activity was measured in fresh muscle homogenate and isolated mitochondria according to the protocol by Srere et al. [23].

Oxygen consumption of isolated mitochondria was measured using a Clark-type oxygen electrode (Strathkelvin, Scotland, UK) with a chamber volume of 1 ml and a temperature of 25 °C as previously described [24]. Daily calibration with 2 mM NADH was routinely conducted prior to measurements. Isolated mitochondria were incubated in a solution containing 80 mmol/l KCl, 50 mmol/l MOPS, 1 mmol/l EGTA, 5 mmol/l KH_2_PO_4_ and 1 mg/ml fatty acid-free bovine serum albumin at pH 7.4. The rate of respiratory capacity was measured using glutamate (10 mM) as substrate and 10 mM adenosine diphosphate (ADP) as stimulus. The ADP-stimulated oxygen consumption (state 3) and the ADP-limited oxygen consumption (state 4) in the respiratory chamber and the ADP/O ratio (ADP added per oxygen consumed) were determined.

All chemicals were purchased from Sigma-Aldrich (USA), Thermo Scientific (USA), Carl Roth (Germany) and Invitrogen (USA).

### 2.6 Western blot and RT-PCR

Protein extraction was performed in ventricles snap frozen and ground under liquid nitrogen with a sample buffer containing proteinase and phosphorylase inhibiting factors (20 mM Tris, 50 mM NaCl, 25 mM NaF, 12.5 mM Na_4_P_2_O_7_, 1% Triton-X, 0.1 M Na_3_VO_4_, protease inhibitor cocktail, 1 mM PMSF). Protein concentrations of homogenized left ventricles were measured using the Bradford method. Approx. 40 μg of protein was loaded onto a 10% polyacrylamide gel and separated at 40 mA for 1 h. After separation, proteins were transferred onto a PVDF membrane (10344661, Merck, USA) and incubated over night at 4 °C with gentle agitation with primary antibodies (concentration 1:1000) against insulin receptor (InsR/IGF-1R) (#3025, Cell Signaling, USA), Phospho-InsR/IGF-1R (#3024, Cell Signaling, USA), Akt (#9272S, Cell Signaling, USA), Phospho-Akt (Ser473) (#9271S, Cell Signaling, USA), total OXPHOS (antibody cocktail detecting one subunit per respiratory complex) (MS604, MitoSciences, USA), GAPDH (ab37168, Abcam, UK). Incubation with secondary antibodies (concentration 1:40000; #NA934V, #NXA931 Amersham, USA) was carried out at room temperature for 1 h. Bands were visualized using chemiluminescence solution (42582, Serva, Germany) and 1% H_2_O_2_ using an LAS4000 imaging system (GE Healthcare, USA), and semi-quantified using ImageJ software in a blinded manner.

The Δ insulin response was calculated for each condition as the fold-change or absolute difference in the relative p-Akt/total Akt ratio or p-IR/total IR between the insulin-stimulated state and the corresponding basal mean. To allow accurate normalization, phosphorylated Akt (p-Akt) and total Akt resp. p-IR and total-IR were analyzed on the exact same membrane. Blots were first probed for phosphorylated antibody, stripped, and subsequently reprobed for unphosphorylated antibody.

RNA extraction from heart tissue was performed using TRIzol® reagent (Invitrogen, USA) after tissue maceration in liquid nitrogen. RNA concentrations were measured using a spectrophotometer (ND-1000, peqlab, Germany). Quantitative real-time PCR was performed using Sensi FAST™ SYBR® No-ROX one-step-kit (BIO-72005, Bioline, USA) according to the manufacturer's instructions. Each reaction contained 1.5 μl of cDNA template, 200 nM of forward and reverse primers, and Master Mix in a final volume of 10 μl. Thermal cycling parameters comprised an initial denaturation at 95 °C for 3 min, followed by 40 cycles of 95 °C for 15 s and 60 °C (58.5 °C) for 30 s. Amplification specificity was verified by post-PCR melt-curve analysis (65-95 °C). Transcript levels were normalized to β-actin levels. Relative fold-changes were calculated using the 2^−ΔΔCt^ method. Primers were purchased from Eurofins (Germany) and the following primer pairs were used:

*Sirt1*-forward ATCTATGCTCGCCTTGCGGT

*Sirt1*-reverse TGTGACACAGAGACGGCTGG

*β-actin*-forward CCCAGATCATGTTTGAGACCT

*β-actin*-reverse CCATCACAATGCCTGTGGTA

### 2.7 Statistical analysis

Data are presented as mean ± SEM. Longitudinal measurements (tolerance tests and body weight curves) were converted into single integrated summary parameters for each biological replicate: Area Under the Curve (AUC, calculated via the trapezoidal rule) for glycemic clearance, and overall net weight change (body weight gain) for growth dynamics. These endpoint summary metrics, as well as terminal tissue parameters, were analyzed by two-way Analysis of Variance (ANOVA) with Genotype and Diet as main factors, including the Genotype × Diet interaction. Normality of data was assessed using the Shapiro-Wilk test. Outlier were identified by Grubb's test. When ANOVA indicated significant main effects or interactions (*P* < 0.05), Tukey's multiple-comparison test was applied for post-hoc pairwise adjustments. All statistical evaluations were conducted using GraphPad Prism.

## 3 Results

### 3.1 Dietary effects in Sirt1^OE^ mice

Sirt1^OE^ and WT mice were assigned randomly to two groups, a control group that received standard diet and an intervention group that received high-fat diet. HFD provided approximately 60% of all calories from fat (Fig. 1A and B). According to the original description, Sirt1^OE^ mice are characterized by a moderate overexpression across different tissues (∼3-fold increase) [17]. We confirmed the previous findings indicating a doubling of Sirt1 transcript expression (Fig. 1C) and protein expression (Fig. 1D) in cardiac tissue. Under non-stressed conditions, Sirt1^OE^ mice were born at a Mendelian ratio and displayed no deviation from regular appearance and physiology including gain of body mass, gross morphology, insulin sensitivity, cardiac contractile function, and mitochondrial respiratory capacity. The individual diet intervention (SD or HFD) was started at the age of 2 months and lasted for 8 weeks (Fig. 1A). In WT mice, we observed a significant increase with HFD of body weight (Fig. 1E and F) and fat mass (Fig. 1G) while heart and lung mass remained unchanged in all groups (Table S1). Sirt1^OE^ mice, like their WT counterparts, gained body mass upon HFD but to a significant lower extent. This was mainly due to lower fat mass.

### 3.2 Sirt1^OE^ mice were protected from the development of glucose and insulin intolerance

First, we set out to test glucose and insulin response of animals. Upon intraperitoneal application of glucose to test for glucose tolerance (GTT), blood glucose levels reached a maximum after 15 min in the SD group and after 30 min in the HFD group (Fig. 2A) and were significantly higher in WT mice receiving a HFD compared to SD controls (Fig. 2B). The time-course of glucose-clearance was delayed in mice of the HFD group compared to control mice, remaining elevated for 120 min after glucose application (Fig. 2A). Together, these data indicate the development of glucose intolerance in HFD-fed WT animals. Sirt1^OE^ mice on SD showed a normal response to GTT similar to WT animals, but a largely preserved glucose tolerance on HFD (Fig. 2A). Specifically, Sirt1^OE^ mice on HFD showed faster glucose clearance; however, lower adiposity may contribute to this effect.

**Fig. 2:**
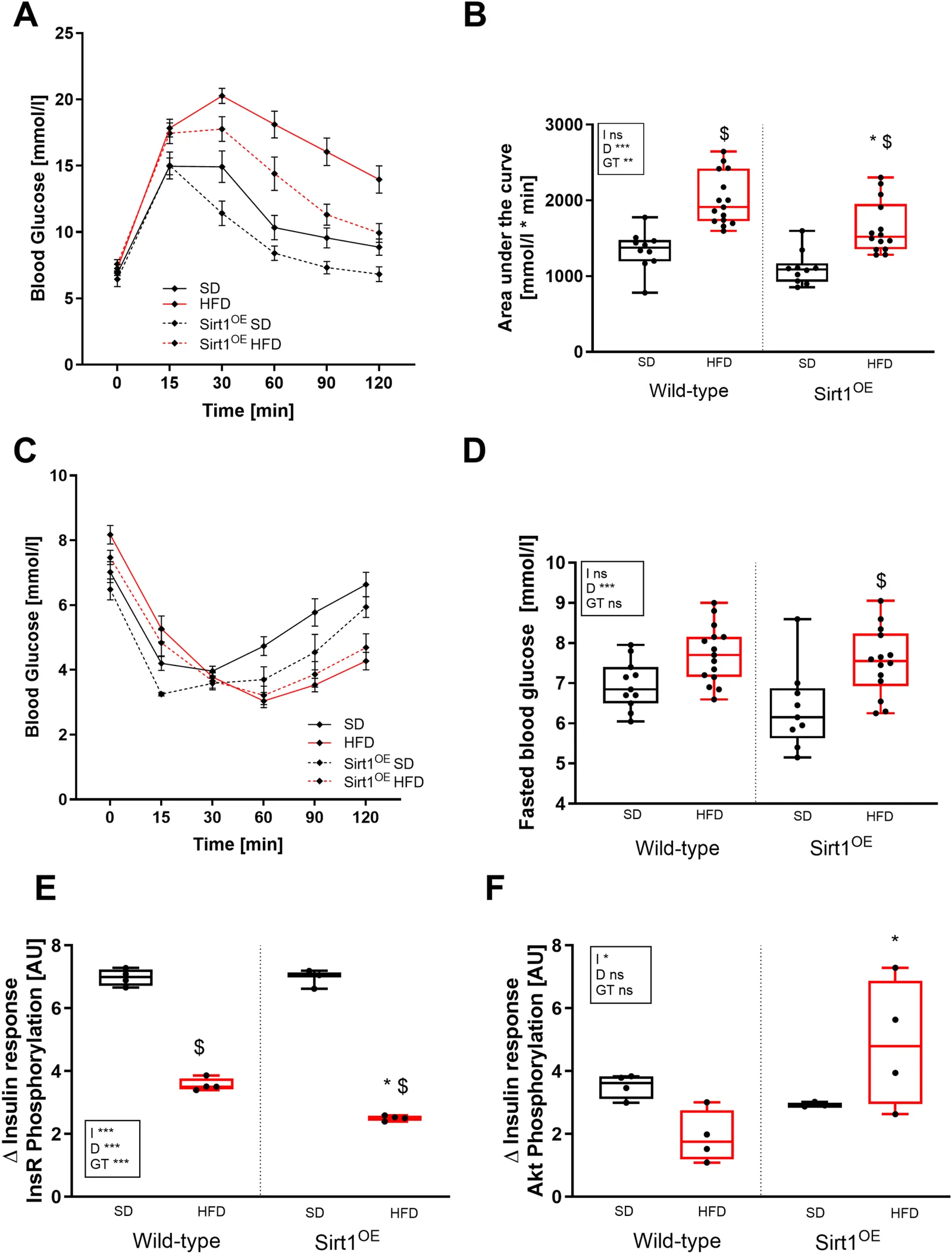
**Whole-body glucose and insulin tolerance and cardiac-specific insulin signaling are disturbed upon 8 weeks of HFD in WT mice but not Sirt1^OE^ mice.** **(**A) Intraperitoneal glucose tolerance test (2 g/kg) (n = 10-15). (**B**) Area under the curve after glucose tolerance test (n = 10-15). (**C**) Intraperitoneal insulin tolerance test (1U/kg) (n = 10-15). (**D**) Fasting blood glucose levels (n = 9-15). (**E**) Ratio of phosphorylated insulin receptor to total amount of insulin receptor (fold change) (n = 3-4). (**F**) Ratio of phosphorylated Akt to total Akt (fold change) (n = 3-4). Data are shown as mean ± SEM or as box-and-whisker plots with individual data points. Two-way ANOVA with Tukey's multi-comparison test was used to test for statistical significance. *P < 0.05, **P < 0.01 and ***P < 0.001 for comparisons as indicated and to wild-type of same diet, and $*P* < 0.05 for comparisons to SD of the same genotype. SD, standard diet; HFD, high-fat diet; Sirt1^OE^, Sirt1 transgenic mice; I, interaction; D, diet; GT, genotype.

Insulin tolerance tests in SD-fed mice (Fig. 2C) showed a steep decline in plasma glucose levels with a minimum already at 15 min after insulin administration and a returning of plasma glucose levels to baseline after 120 min HFD-fed WT mice exhibited altered ITT kinetics characterized by prolonged glucose suppression (∼50% of baseline at 120 min) and delayed counter-regulatory recovery, indicative of impaired systemic glucose homeostasis. Notably, while Sirt1^OE^ mice showed pronounced protection against HFD-induced glucose intolerance (as assessed by GTT), their glycemic response during ITT remained largely similar to HFD-fed WT controls, indicating that Sirt1 overexpression primarily preserves glucose tolerance rather than fully rescuing systemic insulin sensitivity under chronic high-fat feeding. Of note, fasting blood glucose levels were slightly elevated with HFD in both WT and Sirt1^OE^ mice (Fig. 2D).

### 3.3 Sirt1 prevents HFD-induced suppression of cardiac Akt activation downstream of the insulin receptor

Cardiac-specific insulin sensitivity was assessed by insulin-induced phosphorylation of the insulin receptor and its downstream target Akt. Both Akt and insulin receptor protein phosphorylation were decreased with HFD in WT animals (Fig. 2E and F). In Sirt1^OE^ mice, insulin stimulated phosphorylation of the insulin receptor was decreased with HFD comparably to WT animals. However, phosphorylation of Akt, downstream of insulin receptor, remained preserved in Sirt1^OE^ mice with HFD.

### 3.4 HFD induced cardiac diastolic dysfunction was attenuated in Sirt1^OE^ mice

HFD is known to induce a characteristic morphologic remodeling of the heart, hallmarked by impaired ventricle filling but preserved contractility. We determined morphometric and functional parameters by echocardiography (Fig. 3). In WT mice, HFD had no impact on heart rate (Fig. 3A), left ventricular wall thickness (Fig. 3B) and left ventricular end diastolic diameter (Fig. 3C) but led to a decrease in A Wave (Fig. 3D) and a slight increase in E Wave (Fig. 3E) with deceleration time remaining constant (Fig. 3F). Based on these individual parameters, we calculated different established ratios describing diastolic and systolic function of the heart. HFD led to a decrease in diastolic function (Fig. 3G and H) with preserved systolic function (Fig. 3I). Sirt1^OE^ mice displayed normal cardiac morphology (Fig. 3A–C) and contractility (Fig. 3I) on both SD and HFD. Diastolic functional changes induced by HFD were significantly attenuated in the Sirt1^OE^ mice (Fig. 3G and H). This suggests that Sirt1 primarily alleviates the HFD-induced diastolic dysfunction.

**Fig. 3:**
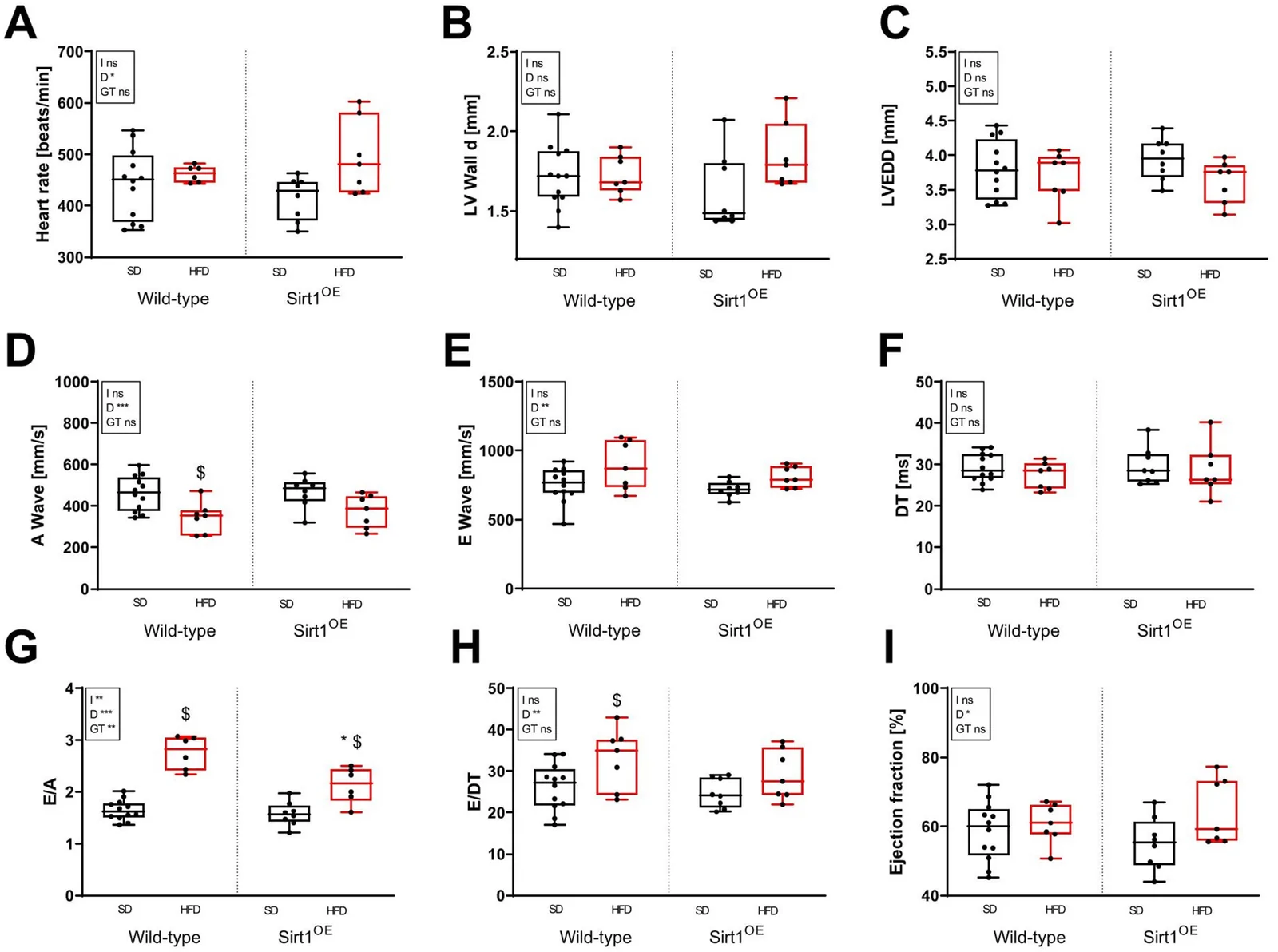
**Diastolic dysfunction upon 8 weeks of HFD is attenuated in Sirt1^OE^ mice.** (**A**) Heart rate (n = 7-12). (**B**) Left-ventricular (LV) wall thickness in diastole (d) (n = 7-12). (**C**) Left ventricular end diastolic diameter (LVEDD) (n = 7-12). (**D**) A-Wave (n = 7-12). (**E**) E-Wave (n = 7-12). (**F**) Deceleration time (DT) (n = 7-12). (**G**) E-Wave – to – A-Wave ratio (E/A) (n = 7-12). (**H**) Ratio E-to-deceleration time (n = 7-12). (**I**) Ejection fraction (n = 7-12). Data are shown as box-and-whisker plots with individual data points. Two-way ANOVA with Tukey's multi-comparison test was used to test for statistical significance. *P < 0.05, **P < 0.01 and ***P < 0.001 for comparisons as indicated and to wild-type of same diet, and $*P <* 0.05 for comparisons to SD of the same genotype. SD, standard diet; HFD, high-fat diet; Sirt1^OE^, Sirt1 transgenic mice; I, interaction; D, diet; GT, genotype.

### 3.5 HFD induced impairment of cardiac respiratory capacity was prevented in Sirt1^OE^ mice

To determine mitochondrial function, citrate synthase activity and mitochondrial respiratory capacities were measured in tissue homogenates and freshly isolated cardiac mitochondria (Fig. 4). The activity of the mitochondrial marker enzyme citrate synthase was unaffected by diet in both WT mice and Sirt1^OE^ mice (Fig. 4A and B). Instead, maximal ADP-stimulated respiratory capacity (state 3 respiration) was significantly decreased in isolated mitochondria of WT mice but not of Sirt1^OE^ mice on HFD (Fig. 4C) with constant ADP-limited (state 4) respiration (Fig. 4D). The absence of a decrease of mitochondrial respiration in Sirt1^OE^ mice on HFD indicates that Sirt1 can protect mitochondria from this HFD-related harmful effect. It remains to be determined what mechanism underlies the observed protection. The mechanism seems not related to mitochondrial complex protein expression, since we did not detect any reduction with HFD in wild-type as in Sirt1^OE^ (Fig. 4E–J).

**Fig. 4:**
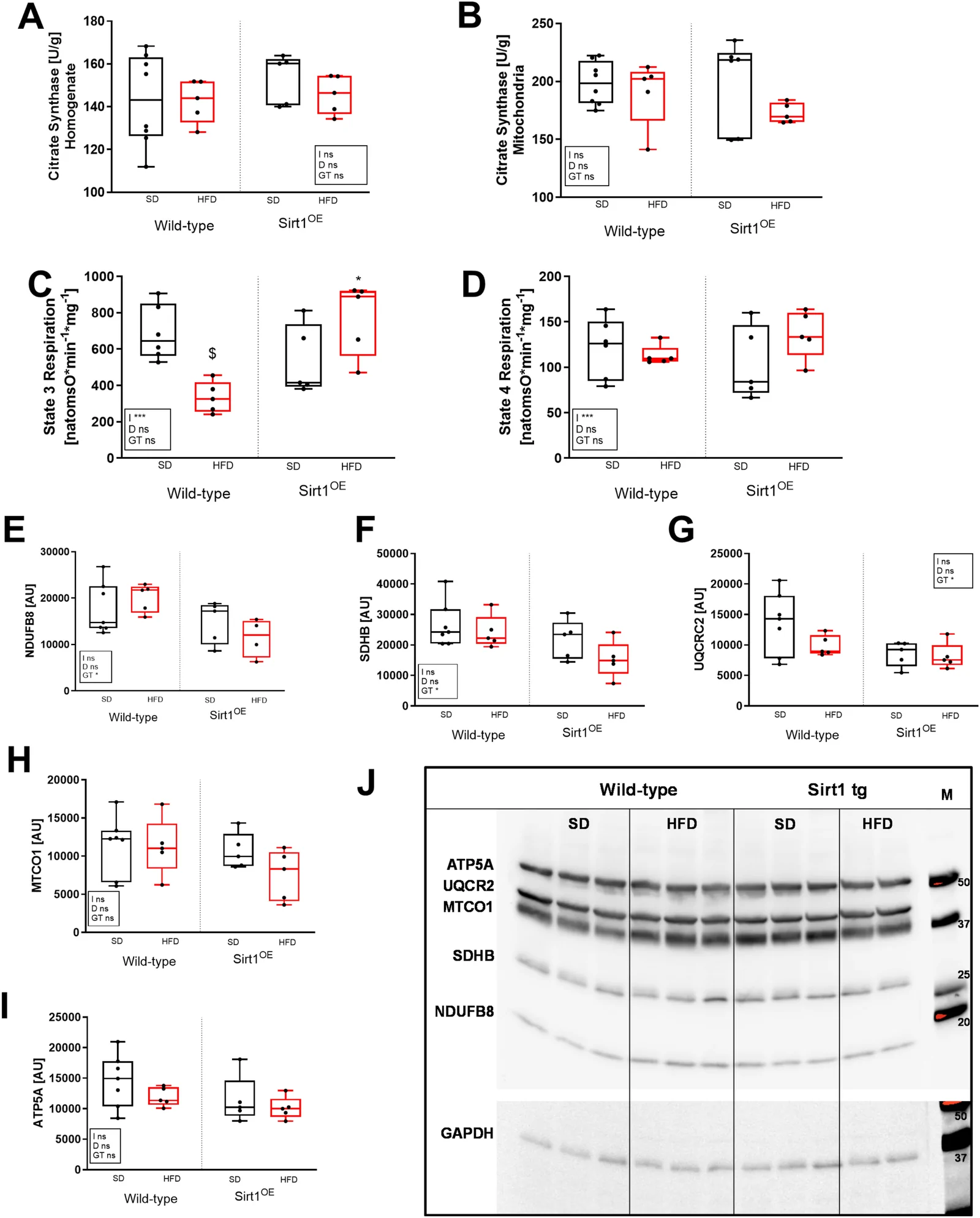
**HFD decreased mitochondrial respiratory capacity in WT mice but not Sirt1^OE^ mice.** (**A**) Citrate synthase activity in cardiac homogenate (n = 5-8). (**B**) Citrate synthase activity in isolated mitochondria (n = 5-8). (**C**) Phosphorylating (state 3) respiration in isolated cardiac mitochondria with glutamate as substrate (n = 5-6). (**D**) ADP-limited respiration (state 4) in isolated cardiac mitochondria with glutamate as substrate (n = 5-6). (**E-I**) Protein expression of selected respiratory chain subunits as indicated (n = 4-7). (**J**) Representative western blots from heart tissue of WT and Sirt1^OE^ upon 8 weeks diet as indicated. Data are shown as box-and-whisker plots with individual data points. Two-way ANOVA with Tukey's multi-comparison test was used to test for statistical significance. *P < 0.05, **P < 0.01 and ***P < 0.001 for comparisons as indicated and to wild-type of same diet, and $*P* < 0.05 for comparisons to SD of the same genotype. SD, standard diet; HFD, high-fat diet; Sirt1^OE^, Sirt1 transgenic mice; I, interaction; D, diet; GT, genotype.

## 4 Discussion

The present investigation determined the extent to which a moderate, ubiquitous overexpression of Sirt1 (Sirt1^OE^) protects against the detrimental cardiac and metabolic consequences of a chronic high-fat diet. Our data demonstrate that Sirt1 plays a pivotal role in modulating mitochondrial phenotype and functional remodeling of the myocardium under metabolic stress. The targeted preservation of Sirt1 expression successfully attenuated or prevented specific pathological alterations, whereas other systemic effects of the HFD persisted.

### 4.1 Sirt1 overexpression does not prevent diabetes mellitus

Although Sirt1 overexpression provided substantial metabolic benefits [25], a detailed analysis of glucose homeostasis revealed that it cannot fully prevent the development of HFD-induced type 2 diabetes mellitus or impaired glucose tolerance. While Sirt1^OE^ mice on a HFD exhibited accelerated glucose clearance during the i.p. glucose tolerance test compared to wild-type controls, both genotypes displayed significantly elevated fasting blood glucose levels under chronic HFD conditions. This discrepancy indicates that Sirt1 primarily enhances peripheral insulin sensitivity and glucose utilization under an acute metabolic challenge, but fails to mitigate the baseline metabolic burden and chronic fasting hyperglycemia - both of which remain key hallmarks of manifest diabetes.

This phenotypical preservation of insulin sensitivity must, however, be interpreted with caution. Although both genotypes gained body mass upon HFD feeding, Sirt1^OE^ mice did so to a significantly lesser extent, driven primarily by a lower accumulation of fat mass. The improved glucose and insulin tolerance curves in the transgenic group may therefore be secondary to their lower adiposity, which independently enhances insulin sensitivity and potentially inflates the apparent direct protection afforded by the transgene. Indeed, literature demonstrates that both fat mass and lean mass independently influence glucose tolerance, and such metabolic benefits frequently disappear when body weight is strictly matched via pair-feeding protocols [26]. Consequently, the isolated advantage in the area under the curve alongside identical fasting hyperglycemia fails to cleanly disentangle the direct cell-autonomous effects of Sirt1 from the indirect systemic benefits of reduced adiposity.

To date, the proposed involvement of Sirt1 in metabolic protection has relied heavily on indirect evidence using small-molecule activators. In particular, beneficial effects of resveratrol in HFD-fed rodents have been widely demonstrated, including improved insulin sensitivity, reduced hepatic steatosis, and increased survival [8,27,28]. More recently, structurally distinct Sirt1 activators have shown promise in improving metabolic function in obese rodents, offering therapeutic potential for type 2 diabetes [29]. However, while these pharmacological results compellingly argue in favor of a protective role for Sirt1, virtually all chemical activators show off target effects and cross-react with other members of the Sirtuin family or affect a diverse range of parallel signaling pathways, as is well-documented for resveratrol [30,31]. In contrast, our investigation addresses this limitation by investigating Sirt1 function using a precise genetic strategy: a BAC transgenic mouse model with a moderate overexpression of Sirt1 under the tight control of its natural promoter. Given that even this highly specific, non-toxic elevation of Sirt1 was insufficient to completely block HFD-induced metabolic dysfunction, these findings indicate that targeted Sirt1 activation alone is insufficient to fully counteract dietary metabolic collapse.

### 4.2 Attenuation of diastolic dysfunction

At the cardiac level, a chronic HFD characteristically induces isolated diastolic dysfunction with a preserved systolic ejection fraction - a hallmark of the HFpEF (heart failure with preserved ejection fraction) phenotype. This clinical picture was reproduced in our HFD-fed wild-type mice, which exhibited marked reductions in A-wave amplitude as well as distinct alterations in E/A and E/DT ratios, defining our model as pre-HFpEF. Transgenic overexpression of Sirt1 significantly mitigated these functional impairments. However, because these diastolic parameters did not fully return to the baseline levels of standard-diet controls, Sirt1 offers a substantial but ultimately partial protection against HFD-induced diastolic dysfunction.

This partial efficacy aligns with recent literature suggesting that while Sirt1 activation acts as a major safeguard against diabetic cardiomyopathy, chronic metabolic stress simultaneously activates secondary, independent pathways. For instance, systemic lipotoxicity triggers advanced glycation end-product accumulation and interstitial fibrosis, which can partially bypass Sirt1-mediated cardioprotection [32,33]. This underlines that while target-specific approaches - such as activating the downstream Sirt1/PGC-1α pathway via rosmarinic acid [34] or modulating the myocardial lipidome via recombinant Sirt1 supplementation [35] - can rescue baseline cardiac function under metabolic stress, they operate within a highly complex network of secondary pathological changes.

Importantly, two major confounding factors prevent us from definitively attributing this partial protection to a direct, cardiac-autonomous action of Sirt1. First, because Sirt1 is overexpressed body-wide, Sirt1^OE^ mice maintained a significantly lower fat mass and improved systemic glucose handling under HFD conditions. This systemic relief naturally reduces preload, afterload, and myocardial metabolic stress, which could secondarily improve ventricular relaxation independently of intrinsic cardiac Sirt1 signaling. Second, a methodological limitation must be considered: diastolic dysfunction was inferred solely from load- and heart rate–dependent transmitral flow indices under isoflurane anesthesia. Since E/A and E/DT ratios are highly sensitive to heart rate, loading conditions, and anesthetic depth [36], these parameters provide a strong indication, but not definitive proof, of altered myocardial relaxation. Consequently, more advanced tissue-level diagnostics, such as speckle-tracking echocardiography or invasive hemodynamic pressure-volume loops, would be required to isolate the direct myocardial contribution of Sirt1.

### 4.3 Protection against mitochondrial dysfunction

In contrast to the partial cardiac protection during HFD, Sirt1 seems to preserve complex I-supported state 3 respiration in isolated cardiac mitochondria. While WT mice on HFD exhibited a significant decline in maximal ADP-stimulated mitochondrial respiration capacity in isolated cardiac mitochondria, this capacity remained fully intact in Sirt1^OE^ mice under given conditions. Given that neither citrate synthase activity nor the protein expression of mitochondrial respiratory chain complexes (Complexes I–V) showed significant alterations, this protective effect does not stem from an increase in mitochondrial mass or altered complex abundance. Instead, it could rely on the functional preservation of mitochondrial respiratory efficiency. This dissociation between respiratory function and complex expression matches established literature: chronic HFD impairs mitochondrial state 3 respiration primarily via oxidative stress, lipotoxic uncoupling, and post-translational hyperacetylation of metabolic enzymes rather than a reduction in organelle density [37,38]. While indirect Sirt1 activation via resveratrol or berberine has been shown to counteract these defects by stimulating mitochondrial autophagy or biogenesis in peripheral tissues [28,39], our model demonstrates that a direct, moderate elevation of Sirt1 protein is sufficient to maintain baseline respiratory efficiency in the myocardium.

This functional preservation of the respiratory chain strongly suggests that Sirt1 maintains upstream cellular survival pathways, preventing the metabolic starvation typically induced by dietary stress. A central molecular explanation for this sustained mitochondrial fitness lies within the downstream insulin signal transduction cascade.

### 4.4 Molecular mechanism: preservation of the Akt response to insulin

Under HFD conditions, WT mice developed a pronounced cardiac insulin resistance, characterized by diminished insulin-induced phosphorylation of the insulin receptor and its downstream signaling effector, Akt. In Sirt1^OE^ mice, the phosphorylation of the insulin receptor was similarly reduced under HFD, yet the downstream activation of Akt (Ser473) remained fully preserved. This selectively maintained Akt response, despite upstream insulin receptor inhibition, suggests that Sirt1 stabilizes alternative signaling pathways or modulates inhibitory proteins (such as PTP1B), thereby keeping the pro-survival and metabolic signaling via Akt intact [40]. Moreover, Sirt1 additionally regulates insulin-induced tyrosine phosphorylation of IRS-2 through deacetylation of this substrate, which affects a crucial step in the insulin signaling pathway [41]. This sustained Akt activity likely secures the metabolic flexibility of the myocardium, directly shielding the mitochondria from the observed respiratory collapse. Our results expand upon established signaling frameworks where Sirt1 acts downstream or independently of the insulin receptor to preserve substrate flexibility. However, while preserved mitochondrial respiration coincided with maintained Akt phosphorylation, Akt signaling was not functionally manipulated in this study. Therefore, the link between Sirt1, Akt activation, and cardiac mitochondrial protection remains observational, and Akt activation should be viewed as a correlated signaling response rather than a proven causal driver.

### 4.5 Limitations of the model

A body-wide BAC transgenic model was utilized to mimic the systemic effects of future pharmacological Sirt1 therapies. However, this global approach introduces two major confounding factors. First, a central finding of our study is that Sirt1^OE^ mice accumulate significantly less body fat during chronic HFD feeding compared to WT controls. This attenuation of adiposity represents a major systemic mechanism that cannot be disentangled from direct cardiac actions in a whole-body transgenic model. Reduced fat accumulation inherently dampens systemic metabolic dysfunction, lowers circulating free fatty acids, and reduces cardiac lipid overload (lipotoxicity), thereby preserving cardiac loading conditions and mitochondrial respiratory integrity. Future studies utilizing cardiomyocyte-specific models or pair-feeding protocols seem mandatory to localize the precise site of Sirt1 action. Second, while the BAC model is a well-characterized functional gain-of-function model (19), we did not directly measure cardiac Sirt1 deacetylase activity or the acetylation status of its canonical targets (e.g., PGC-1α, FOXO1). Given that HFD depletes intracellular NAD + levels - the rate-limiting co-substrate for Sirt1 - increased protein abundance does not automatically guarantee a proportional increase in in vivo enzymatic activity. Nevertheless, the robust downstream preservation of Akt phosphorylation and mitochondrial state 3 respiration strongly suggests that functional Sirt1 signaling was maintained.

Methodological considerations regarding the transgenic construct should also be noted. The BAC-based Sirt1 transgenic mouse line utilized in this study yields a consistent ∼2- to 2.5-fold elevation of Sirt1 protein levels in cardiac tissue, preserving physiological signaling pathways without non-specific overexpression toxicity. While BAC vectors inherently contain adjacent genomic segments, such as Dnajc12, Pfluger et al. previously demonstrated in their characterization of this exact model under HFD conditions that Dnajc12 does not influence the metabolic phenotype and can be considered negligible [17]. Nevertheless, as with any whole-body transgenic setup, future studies utilizing cell-type-specific gene editing will further refine our understanding of cardiac-restricted Sirt1 actions.

In summary, moderate transgenic Sirt1 overexpression driven by its endogenous promoter attenuated HFD-induced complex I respiratory dysfunction, while offering partial protection against glucose intolerance and early diastolic impairment. This dissociation suggests that distinct metabolic and cardiac mechanisms govern these cellular outcomes.

Notably, the 8-week HFD protocol models an early pre-HFpEF phenotype—characterized by preserved LVEF and diastolic filling alterations in the absence of overt concentric hypertrophy. Investigating this early cardiometabolic window demonstrates that Sirt1 preservation can intercept mitochondrial signaling before irreversible structural remodeling occurs. Because Sirt1 in non-cardiac tissues regulates hepatic gluconeogenesis, adipose lipolysis, and central energy homeostasis, the observed cardiac benefits may be partly secondary to improved systemic substrate clearance, adipokine balance, and preserved peripheral insulin sensitivity. Thus, while systemic Sirt1 elevation preserves cardiac insulin signaling and mitochondrial capacity in pre-HFpEF, tissue-specific models will be essential to delineate the precise cell-autonomous contributions of cardiac Sirt1.

## CRediT authorship contribution statement

**Andrea Schrepper:** Writing – review & editing, Writing – original draft, Project administration, Methodology, Investigation, Conceptualization. **Michael Schwarzer:** Writing – review & editing, Conceptualization.

## Ethics declaration

This study was conducted in accordance with the ARRIVE (Animal Research: Reporting of In Vivo Experiments) guidelines. This study was approved by the Thüringer Landesamt für Verbraucherschutz. (Approval No. 02-081/14)

## Ethical statement

This study was conducted in accordance with the ARRIVE (Animal Research: Reporting of In Vivo Experiments) guidelines.The experimental protocols were approved by the local authorities (Thüringer Landesamt für Verbraucherschutz, approval ID 02-081/14).

## Data access statement

Access to the data that support the findings of this study is restricted. However, data may be available from the corresponding authors upon reasonable request.

## Declaration of competing interest

The authors declare that the research was conducted in the absence of any commercial or financial relationships that could be construed as a potential conflict of interest.
