# Supplementary material for: Ubiquitous Sirt1 overexpression is associated with preserved cardiac diastolic function and mitochondrial respiratory capacity in mice on a high-fat diet
Source: Metabol Open. 2026 Sep 29;32:100503. doi: 10.1016/j.metop.2026.100503 (PMC13672833; doi:10.1016/j.metop.2026.100503)
Supplement: Supplementary file 1 [file mmc1.docx]

**Table S1. Morphological and echocardiographic parameters. Data are shown as mean ± SEM.** Two-way ANOVA was used to test for statistical significance. **P* < 0.05, ***P* < 0.01 and ****P* < 0.001 for comparisons as indicated, and $*P* < 0.05 for comparisons between the same genotypes. SD, standard diet; HFD, high-fat diet; Sirt1^OE^, Sirt1 transgenic mice; I, interaction; D, diet; GT, genotype.

|  |  | WT | | Sirt1^OE^ | |  |  |  |
| --- | --- | --- | --- | --- | --- | --- | --- | --- |
|  |  | SD | HFD | SD | HFD | I | D | GT |
| Body mass | [g] | 27.7 ± 0.5 | 33.1 ± 0.9^$^ | 27.0 ± 0.5 | 30.4 ± 0.7^*$^ | ns | *** | * |
| Heart mass | [mg] | 126 ± 3 | 124 ± 3 | 130 ± 3 | 125 ± 2 | ns | ns | ns |
| Lung mass | [mg] | 148 ± 3 | 155 ± 5 | 152 ± 6 | 154 ± 3 | ns | ns | ns |
| Liver mass | [mg] | 1482 ± 38 | 1595 ± 63 | 1407 ± 35 | 1460 ± 41 | ns | ns | * |
| E/E’ |  | 26,1± 1,8 | 30,6 ± 1,7 | 25,0 ± 1,1 | 31,1 ± 1,7 | ns | ** | ns |

**Table S2. Respiratory control index and ADP/O ratio of isolated mitochondria with substrate glutamate. Data are shown as mean ± SEM.** Two-way ANOVA was used to test for statistical significance. **P* < 0.05, ***P* < 0.01 and ****P* < 0.001 for comparisons as indicated and to wild type of same diet, and ^$^*P* < 0.05, ^$$^*P* < 0.01 and ^$$$^*P* < 0.001 for comparisons between the same genotypes. SD, standard diet; HFD, high-fat diet; Sirt1^OE^, Sirt1 transgenic mice; I, interaction; D, diet; GT, genotype.

|  |  | WT | | Sirt1^OE^ | |  |  |  |
| --- | --- | --- | --- | --- | --- | --- | --- | --- |
|  |  | SD | HFD | SD | HFD | I | D | GT |
| RCI |  | 5.90 ± 0.57 | 4.31 ± 1.08^$^ | 5.23 ± 0.34 | 5.69 ± 0.56^*^ | * | ns | ns |
| ADP/O |  | 0.91 ± 0.06 | 1.37 ± 0.10^$$^ | 0.82 ± 0.04 | 1.21 ± 0.13^$$^ | ns | *** | ns |

**Figure S1. Sirt1 transcript and protein level in cardiac tissue.**

(**A**) *Sirt1* transcript level normalized to *β-actin* in mice as indicated (n = 4). (**B**) Cardiac Sirt1 protein expression (n = 4). Data are shown as box-and-whisker plots with individual data points. Two-way ANOVA with Tukey`s multi-comparison test was used to test for statistical significance. *P < 0.05. SD, standard diet; HFD, high-fat diet, Sirt1^OE^; Sirt1 transgenic mice.

**Figure S2. Western blots relating to Fig. 2E, F.**

SD, standard diet; HFD, high-fat diet; Sirt1^OE^, Sirt1 transgenic mice; WT, wild-type littermates; +I, + insulin; -I, without insulin; IR, insulin receptor; P-IR, phosphorylated insulin receptor; Akt, protein kinase B; P-Akt, phosphorylated protein kinase B.

Supplementary material – uncropped Western blots

Oxphos	GAPDH

MitoScience MS604 1:1000	Abcam ab37168 1:1000

Phospho-Akt	Akt

Cell Signaling 9271s 1:1000	Cell Signaling 9272S 1:1000

Phospho-Insulin Receptor	Insulin Receptor

Cell Signaling 3024s 1:1000	Cell Sign. 3025 1:1000

Sirt1

Cell Signaling 8469s 1:1000
